# The patterns of rehabilitation service use and personal recovery among persons with psychiatric disability

**DOI:** 10.1192/j.eurpsy.2023.696

**Published:** 2023-07-19

**Authors:** L.-Y. Song

**Affiliations:** Graduate Institute of Social Work, National Chengchi University, Taipei, Taiwan, Province of China

## Abstract

**Introduction:**

Rehabilitation services are supposed to facilitate recovery. However, there is no concrete evidence in Taiwan.

**Objectives:**

This study examined the patterns of rehabilitation service use and the association between the pattern of use and personal recovery.

**Methods:**

Thirty-two community psychiatric rehabilitation centers in Taiwan agreed to participate in this study. A sample of 592 participants filled out the questionnaires. Eight kinds of rehabilitation services were included: Independent living and self-care training, interpersonal and social skills training, daily life arrangement and community life rehabilitation, physical activities, symptom management training, occupational therapy, sheltered workshops, and vocational training. Recovery was measured by the Stage of Recovery Scale. Cluster analysis was utilized to classify service use patterns among the participants. ANOVA was used to examine the association between the pattern of use and recovery.

**Results:**

The results revealed five patterns of use: (1) Overall middle level with emphasis on work, (2) independent living plus occupational rehabilitation, (3) independent living plus vocational rehabilitation, (4) overall low-level of use, and (5) overall high-level of use. The differences among the five groups of participants in each kind of rehabilitation service were significant (Eta2=19.2%). The recovery status of overall high users was significantly better than middle-level and low users. The recovery status of low-level users was significantly worse than the other four groups.

**Conclusions:**

The findings imply that greater rehabilitation service use is conducive to recovery. Comprehensive use of various types of service or the combination of independent living and other types seem to facilitate recovery.

**Disclosure of Interest:**

None Declared

